# 

**Published:** 1916-09-16

**Authors:** 


### CONTENTS.

PAGE
**County Councils and Public Health** ... ... 551
**The Clean Milk Campaign** ... ... ... ... 552
**Hospital and Institutional News** ... ... ... 553
The Queen and the London School of Medicine for Women—British Red Cross Stamp—The War and the Medical Defence Union—An Assistant Medical Officer's Peerage—Two Rogues Sentenced—Alleged Charges to Wounded in Hospital—Sir John Furley's New Suggestion—Medical Arrangements in Mesopotamia—The Freemasons' War Hospital—Are Australians Unhappy in Birmingham Hospitals?—Cambridge and District Workers' Hospital Fund—The Doctor's Bills of Public Officials—Wounded Soldiers' Pocket-money—Rapid Action under War Charities Act—Fatal Explosion at an Infirmary—The Staffing of Military Hospitals—The Recovery of the Schools—The Founder of the Oxford Eye Hospital—Medical Treatment of Soldiers' Dependants—The Future of Winsley Sanatorium—An Old Grievance Restated—The German Hospital Report—The Medical Superintendentship of Claybury—The Detection of Tuberculosis Contact Cases—The Local Government Board and a Medical Officer's Suspension—A Testator's Thought for Nurses—Patients and Cases in Hospital Gazettes—This Week's Drug Market.
**Neurasthenia** ... ... ... ... 559
A Distinct and Specific Pathological Condition.

PAGE
**A Federal Report on Venereal Diseases** ... 560
Drastic Proposals.
**National Insurance Act Developments...** ... 561
The New Unemployment Insurance Act.
**The Matrons' and Sisters' Department** ... 563
The College of Nursing.
The Future of British Nursing.
Mental Nurses in Military Hospitals.
**The Editor's Letter-Box** ... ... ... ... ... 565
The Notification of Venereal Diseases—Patients and Nurses: Complaints of Familiarity—Twilight Anæsthesia in Labour.
**Hospital and Institutional Results in 1915** ... 566
**Macedonia in War-Time** ... ... ... ... 567
Its Country and Inhabitants.
**Hospital Architecture and Construction** ... 568
Royal United Hospital, Bath.
**The War Charities Act, 1916** ... ... ... 569
Charity Commission's Memorandum.
**The Book Market** ... ... ... ... ... ... 569
**Matrons' and Sisters' Appointments** ... ... 570
**Passing Events and Topics...** ... ... ... 570
**Doctors and Friendly Societies in New Zealand** ... ... ... ... ... ... 570
**Personalia...** ... ... ... ... ... ... ... 570
**The Institutional Worker** ... ... (Suppl.) 1

